# Effect of Oral Glucose Administration on Ghrelin Levels in Normal-Height Prepubertal Children Born Small for Gestational Age (SGA)

**DOI:** 10.3390/ijms27041791

**Published:** 2026-02-13

**Authors:** Anna Fedorczak, Paula Smalczewska, Magdalena Grobelna, Małgorzata Szałapska, Arkadiusz Zygmunt, Renata Stawerska

**Affiliations:** 1 Department of Endocrinology and Metabolic Diseases, Polish Mother’s Memorial Hospital—Research Institute, 93-338 Lodz, Poland; 2 Department of Pediatric and Adult Endocrinology, Medical University of Lodz, 93-338 Lodz, Poland

**Keywords:** ghrelin, SGA, OGTT, prepubertal children, obesity, metabolic syndrome

## Abstract

Ghrelin is a regulator of appetite and growth hormone secretion, rising during fasting and decreasing after food intake. Children born small for gestational age (SGA) who undergo postnatal catch-up growth are at increased risk of obesity and metabolic disturbances, which may be related to impaired ghrelin regulation. This study assessed fasting and post-glucose ghrelin responses during an oral glucose tolerance test (OGTT) in prepubertal SGA children in relation to obesity and metabolic syndrome components. Ninety-eight children aged 5–9 years were included. Anthropometry, blood pressure, fasting lipids, glucose, insulin, and ghrelin were measured; BMI SDS and HOMA-IR were calculated. Ghrelin concentrations were assessed at baseline and 120 min after glucose ingestion. Ghrelin levels declined significantly during OGTT. Obese SGA children showed lower fasting and post-load ghrelin levels and a smaller decline compared with non-obese peers. Fasting and post-load ghrelin correlated inversely with BMI SDS, waist circumference, and insulin levels. In regression analyses, fasting ghrelin and its suppression were independently associated with HOMA-IR, whereas post-load ghrelin was determined by post-load insulin. These findings indicate that ghrelin dynamics in SGA children are more closely related to insulin sensitivity than adiposity and are blunted in the presence of obesity.

## 1. Introduction

Ghrelin, a hormone produced predominantly by X/A-like cells in the gastric oxyntic mucosa and secreted into the circulation, stimulates orexigenic centers to promote food intake and energy storage, reduces energy expenditure and lipolysis, and also stimulates growth hormone (GH) secretion from the pituitary gland [1,2]. Its secretion fluctuates with the nutritional status—rising during fasting and falling after food ingestion—thereby contributing to energy balance and appetite regulation. However, circulating ghrelin concentrations have been shown to inversely correlate with adiposity. Studies have demonstrated that obese individuals exhibit lower fasting ghrelin levels compared with lean subjects [3,4]. Moreover, it appears that ghrelin may have a greater effect on postprandial satiety than on preprandial appetite stimulation [5]. On the other hand, studies evaluating the effects of a low-glycemic index (GI) diet on ghrelin secretion and satiety have shown that subjective satiety is higher after low-GI meals, whereas postprandial ghrelin levels are higher or not significantly more suppressed compared with high-GI meals [6].

Children born small for gestational age (SGA)—most commonly defined as having a birth weight and/or length below the 10th percentile, the 3rd percentile, or more than 2 standard deviations below the mean for gestational age (GA)—exhibit distinct developmental trajectories characterized by altered early growth patterns and long-term metabolic vulnerability [7]. Many SGA children exhibit rapid catch-up growth and body mass during infancy and early childhood [7]. Although the catch-up growth is usually beneficial for achieving normal stature, disproportionate catch-up weight may predispose them to excessive fat accumulation, insulin resistance, and the early onset of metabolic syndrome features [8]. These developmental characteristics suggest that regulatory pathways involved in appetite control, energy homeostasis, and growth may be altered early in life in individuals born SGA. However, the hormonal mechanisms linking impaired fetal growth, postnatal growth patterns and subsequent metabolic risk remain incompletely understood. Assessment of dynamic hormonal responses to metabolic stimuli may provide additional insight into these mechanisms beyond fasting measurements alone. Oral glucose tolerance testing (OGTT) represents a standardized physiological model of postprandial metabolic challenge, enabling evaluation of glucose-induced hormonal regulation under controlled conditions. This approach is particularly informative in children born SGA, in whom early alterations in insulin sensitivity and postprandial metabolism may be present [9].

Given the central role of ghrelin in appetite regulation, energy metabolism, and GH secretion, it is plausible that altered ghrelin secretion patterns contribute to the distinct metabolic and growth profiles observed in SGA individuals [10]. We hypothesize that abnormal ghrelin dynamics may be associated with increased weight gain and early metabolic alterations in SGA children. Therefore, the present study aimed to assess fasting ghrelin levels and the degree of its reduction during OGTT in prepubertal SGA children in relation to BMI status and the presence of metabolic syndrome components.

## 2. Results

### 2.1. Study Group Characteristics

The study included 98 SGA children with catch-up growth (59% girls). The median age of the children was 7.0 (5.9–8.2) years. All children were in the prepubertal period, defined as Tanner stage I. Thirty-nine children (40%) were obese (defined as BMI SDS ≥ 2.0). Obese children were older and had higher height, height SDS, BMI, BMI SDS and waist circumference than non-obese peers (*p* < 0.001 for all). These differences reflect normal age-related increases in height and body mass during childhood, as well as the rise in obesity prevalence with advancing age.

The groups did not differ significantly in sex distribution (*p* = 0.97). Birth weight, gestational age, and birth weight SDS were also comparable between groups. Study group characteristics divided by obesity status is presented in Table 1.

### 2.2. Results of OGTT in SGA Children

At baseline (0′, prior to oral glucose administration), fasting glucose concentrations did not differ significantly between obese and non-obese SGA children. None of the participants met the criteria for diabetes mellitus. Impaired fasting glucose (≥100 mg/dL) was found in four children, while impaired glucose tolerance (≥140 mg/dL at 120′ of OGTT) was observed in four participants. Glucose concentrations at 60 min exceeding 155 mg/dL were noted in 13 children, but in 9 of them, the overall glucose responses (after 120′) remained within the normal range. Median glucose concentrations at 0′, 60′ and 120′ did not differ significantly between obese and non-obese children [82 (75–88) vs. 83 (80–86) mg/dL, *p* = 0.50; 106 (91–126) vs. 107 (87–132) mg/dL; and 106 (98–113) vs. 102 (91–115) mg/dL, *p* = 0.41, respectively]. In contrast, insulin levels were markedly higher in the obese subgroup at all time points [2.22 (1.50–4.39) vs. 4.56 (2.28–7.56) µIU/mL, *p* = 0.0003 at 0′; 12.5 (8.41–19.4) vs. 24.6 (11.5–35.7) µIU/mL, *p* = 0.004; and 12.0 (8.94–18.3) vs. 25.2 (16.5–38.1) µIU/mL, *p* < 0.001 at 120′]. Consequently, HOMA-IR was significantly higher in obese compared with non-obese participants [0.90 (0.47–1.55) vs. 0.46 (0.30–0.97), *p* = 0.0003], indicating higher insulin resistance.

**Figure 1:**
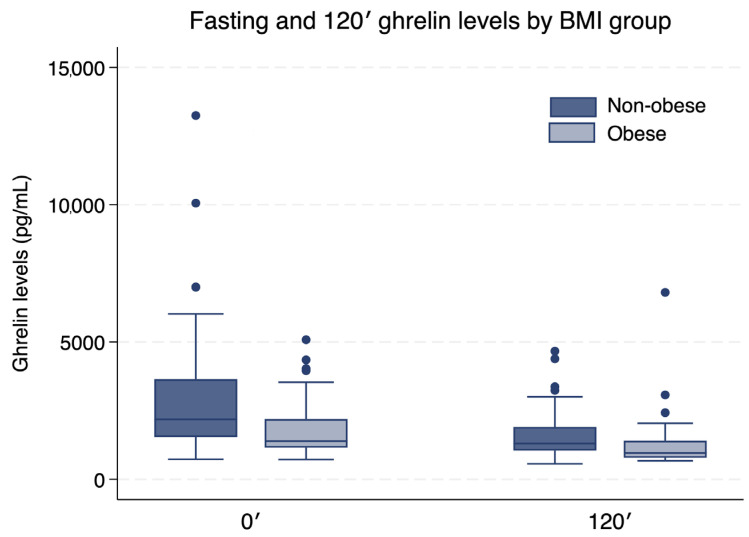
Changes in serum ghrelin concentrations during the OGTT in normal-height prepubertal children born SGA.

Ghrelin concentrations decreased significantly during OGTT in the whole study group [2182 (1538–3648) vs. 1303 (1046–1908) pg/mL at 0′ and 120′, respectively; *p* < 0.001]. In obese children (BMI SDS > 2.0), fasting ghrelin levels were significantly lower than in non-obese peers [1389 (1153–2191) vs. 2182 (1538–3648) pg/mL; *p* = 0.0007], and the decline during the test (Δ0–120′) was significantly smaller [422 (146–789) vs. 689 (370–1597) pg/mL; *p* = 0.0026]. At 120 min after glucose ingestion, ghrelin levels remained lower in obese compared with non-obese participants [957 (781–1402) vs. 1303 (1046–1908) pg/mL; *p* = 0.0046]. These results are summarized in Table 2 and illustrated in Figure 1.

Abdominal obesity, defined as a waist circumference above the 90th percentile for sex and age, was observed in 20 children. Ghrelin levels were significantly lower in children with increased waist circumference compared with those with normal waist circumference, both at baseline and at 120 min during OGTT [1901 (1356–3532) vs. 1407 (1148–2323) pg/mL, *p* = 0.04; and 1272 (966–1772) vs. 1013 (757–1294) pg/mL, *p* = 0.03, respectively], whereas there was no significant difference in ghrelin decline between the groups.

No participants met the criteria for insulin resistance (HOMA-IR > 2.5). High HOMA-IR, defined as values above the 75th percentile (1.2), was found in 46% of obese SGA children (BMI SDS > 2) and in 10% of non-obese children (*p* < 0.001). Given the discrepancies in the interpretation of HOMA-IR values reported in the literature [11], SGA children were also divided into lower and higher HOMA-IR groups by splitting the cohort into two equal halves. Children with higher HOMA-IR compared with those with lower HOMA-IR had lower fasting ghrelin concentration [1424.9 (1148.8–2726.4) vs. 2102.2 (1539.6–3874.5) pg/mL, *p* = 0.006], 120 min ghrelin concentration [1068.6 (766.6–1592.7) vs. 1287.0 (1106.1–1873.4) pg/mL, *p* = 0.02] and the overall decline during OGTT [438.5 (166.2–937.2) vs. 685.1 (381.4–1732.6) pg/mL, *p* = 0.009].

### 2.3. Correlations Between Ghrelin and Metabolic Parameters

**Figure 2:**
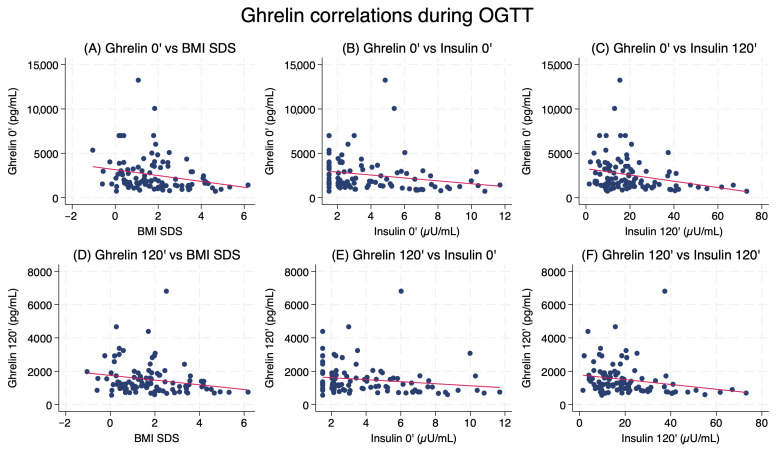
Correlations between fasting and post-load ghrelin levels and metabolic parameters during OGTT in children SGA. Panels (**A**–**C**) show relationships for ghrelin 0′; panels (**D**–**F**) for ghrelin 120′. The red line indicates the linear regression fit.

**Figure 3:**
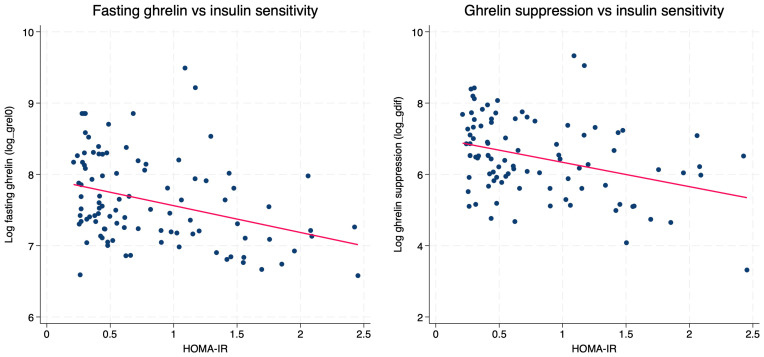
Associations between ghrelin measures and HOMA-IR in SGA children. The red line indicates the linear regression fit.

Fasting ghrelin levels correlated negatively with BMI SDS (*r_s_* = −0.29, *p* = 0.004), waist circumference (*r* = −0.27, *p* = 0.007), HOMA-IR (*r* = −0.21, *p* =0.036), fasting insulin (*r* = −0.36, *p* < 0.001), and insulin levels at 120 min (*r* = −0.34, *p* < 0.001). Similarly, ghrelin levels measured at 120 min after glucose load showed inverse correlations with BMI SDS (*r* = −0.29, *p* = 0.004), waist circumference (*r_s_* = −0.29, *p* = 0.004), fasting insulin (*r_s_* = −0.31, *p* = 0.002), and insulin at 120 min (*r_s_* = −0.40, *p* < 0.001) (Figure 2). Ghrelin suppression (Δ ghrelin) also demonstrated significant negative correlations with BMI SDS (*r* = −0.29, *p* = 0.005), waist circumference (*r* = −0.28, *p* = 0.007), fasting insulin (*r* = −0.33, *p* = 0.001), 120 min insulin (*r* = −0.33, *p* = 0.001), and HOMA-IR (*r* = −0.35, *p* = 0.001), indicating that a reduced decline in ghrelin during OGTT is associated with higher adiposity and impaired insulin sensitivity (Figure 3). No significant correlations were observed between ghrelin concentrations (fasting, 120 min, or Δ ghrelin) and glucose or insulin levels measured at 60 min during OGTT (all *p* > 0.05).

**Figure 4:**
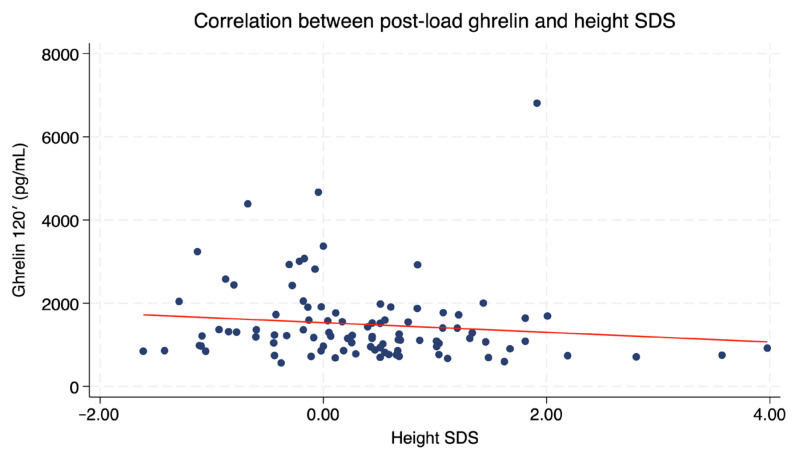
Correlation between post-load ghrelin and height SDS. The red line indicates the linear regression fit.

Post-load ghrelin levels correlated negatively with Height SDS (*r* = −0.23, *p* = 0.026, Figure 4), while fasting and Δ ghrelin levels showed no significant relationships with height.

### 2.4. Ghrelin Levels and Blood Pressure

Elevated blood pressure, defined as values exceeding the 90th percentile for sex and height, was identified in 39 participants. No significant correlations were observed between fasting, post-load, or Δ ghrelin levels and systolic or diastolic blood pressure. Ghrelin concentrations did not differ between groups divided according to high and normal blood pressure. Systolic blood pressure correlated positively with age, adiposity indices (BMI, BMI SDS, waist circumference), fasting and post-load insulin, and HOMA-IR (all *p* < 0.05). Diastolic blood pressure was also positively associated with age and adiposity parameters.

### 2.5. Ghrelin Levels and Lipid Profile

Fasting ghrelin levels showed no significant associations with total, HDL, LDL cholesterol, or triglycerides. Similarly, post-load (120′) ghrelin levels and the change in ghrelin during OGTT (Δ0–120′) were not significantly correlated with any of the lipid parameters. Ghrelin concentrations did not differ between groups with higher and lower lipid parameter concentrations.

### 2.6. Determinants of Fasting, Post-Load, and Suppressed Ghrelin Responses in SGA Children

Higher BMI SDS was associated with lower fasting ghrelin levels (β = −0.13, *p* = 0.003), accounting for approximately 9% of its variance. HOMA-IR showed an even stronger inverse association (β = −0.38, *p* = 0.001; 12% explained variance). In the fully adjusted model including BMI SDS, HOMA-IR, age, and sex, only HOMA-IR remained a significant independent predictor of fasting ghrelin (β = −0.53, 95% CI −0.96 to −0.10, *p* = 0.017), whereas BMI SDS was no longer significant (*p* = 0.266), indicating that fasting ghrelin is more closely related to insulin sensitivity than to adiposity.

Similarly, post-load ghrelin was independently determined by 120 min insulin, whereas BMI SDS, age, and sex were not significant in the multivariable model. Ghrelin suppression in multivariable regression was independently predicted by HOMA-IR (β = −0.28, 95% CI −0.51 to −0.04, *p* = 0.0217), whereas BMI SDS and insulin120′ were not significant.

## 3. Discussion

Ghrelin is an essential metabolic hormone involved in appetite control, energy balance, and growth hormone secretion [1,12]. It circulates predominantly in its unacylated form, whereas the acylated, bioactive form requires modification by ghrelin O-acyl transferase (GOAT) [12]. By binding to the functional splice variant of the growth hormone secretagogue receptor (GHSR-1a), this bioactive form triggers the downstream pathways that mediate ghrelin’s metabolic and endocrine functions [1]. Ghrelin levels vary with age, being higher in childhood and decreasing during puberty [13]. Ghrelin secretion follows a circadian rhythm—rising nocturnally and declining in the morning—and fluctuates during the day in response to fasting and food intake [1]. It is typically elevated in states of energy deficit and suppressed in positive energy balance, including obesity [1,4,14]. Higher BMI is associated with reduced nocturnal ghrelin secretion [15]. Rather than driving weight change directly, ghrelin functions as part of the homeostatic system that regulates energy balance [12]. Obesity is associated with impaired central (NPY/AgRP) responsiveness to circulating ghrelin, resulting in a blunted neuroendocrine ghrelin response that limits further food intake [16].

Lower fasting ghrelin levels and impaired postprandial suppression have consistently been reported in obese children [17,18,19]. Both acylated and unacylated ghrelin were reduced in obesity, with a relatively higher proportion of acylated ghrelin observed in individuals with higher BMI [17]. Ghrelin levels were negatively associated with excess weight and insulin secretion [18]. In our earlier work, we observed a similar pattern in children with short stature, where ghrelin concentrations varied according to BMI—being higher in lean children and lower in those with obesity [20]. In obese children, the decline in plasma ghrelin levels following oral glucose administration was markedly blunted—about a 28% reduction from baseline—compared to the about 60% decrease in normal adults and 50% in anorexic patients [19]. On the other hand, there was no rapid fall in plasma levels of acylated ghrelin in obese children after OGTT at 30 min, but there was an increase at 120 min [21].

Evidence on ghrelin dynamics in SGA children remains limited and comes only from infancy [22]. SGA neonates exhibit elevated ghrelin concentrations, which may facilitate early catch-up growth, and infants with higher ghrelin levels tend to gain weight more rapidly [5,23]. After glucose administration, ghrelin levels decreased rapidly, but infants with greater weight gain showed a notably reduced suppression [5]. However, data in older children are scarce, and the effect of impaired fetal growth on later ghrelin responses—particularly during metabolic challenges such as OGTT—is not well understood. Our earlier work showed elevated ghrelin and IGF-I concentrations in normal-height SGA children who achieved catch-up growth, suggesting that ghrelin contributes to the compensatory endocrine adaptations characteristic of the SGA phenotype [24].

To our knowledge, this is the first study to characterize ghrelin secretion pattern in SGA children after catch-up growth. We found that ghrelin levels decreased significantly during OGTT in the entire cohort of SGA children, with lower fasting and 120 min levels and a smaller decline observed in obese children. This pattern aligns closely with findings from obesity research, where fasting ghrelin is consistently reduced and postprandial suppression is blunted due to impaired enteroendocrine signaling and central satiety resistance [19,25,26]. Furthermore, children with higher HOMA-IR had lower fasting ghrelin, lower 120 min ghrelin, and a smaller overall decline during OGTT compared with those with lower HOMA-IR. No associations were observed between ghrelin levels and other components of the metabolic syndrome, including blood pressure or lipid parameters.

We found that fasting ghrelin concentrations correlated with insulin sensitivity (reflected by HOMA-IR), insulin levels, and BMI SDS; however, multivariable analyses indicated that ghrelin was independently linked to HOMA-IR but not to BMI SDS. Furthermore, the magnitude of ghrelin suppression during OGTT showed the same relationship. These findings are consistent with mechanistic data indicating that insulin directly suppresses ghrelin secretion and indicate that fasting ghrelin more accurately reflects insulin sensitivity than to adiposity in SGA children [27]. A similar pattern was observed for post-load ghrelin: although 120 min ghrelin correlated with BMI SDS in univariate analyses, only 120 min insulin remained an independent predictor in multivariable models. This suggests that the apparent influence of adiposity on postprandial ghrelin is mediated principally through insulin resistance rather than body mass itself. A comparable link between ghrelin and insulin resistance has been described in obese and overweight individuals [28,29].

A striking observation in our cohort was the high prevalence of obesity (40%) and substantially exceeding rates reported in the general Polish pediatric population of similar age [30]. Additionally, a considerable proportion fulfilled the diagnostic criteria for metabolic syndrome, despite being below the age threshold for its formal diagnosis [31]. This is consistent with extensive evidence that SGA-born children are more prone to developing endocrine–metabolic abnormalities than children born AGA [32,33]. Numerous studies have linked SGA status to increased long-term risk of insulin resistance, dyslipidemia, hypertension, and type 2 diabetes mellitus, particularly in the presence of excessive catch-up growth or later-life obesity [9,34]. Our findings also align with the “thrifty phenotype” hypothesis, which proposes that fetal undernutrition induces adaptive metabolic responses that promote survival early in life but may become maladaptive in energy-rich postnatal environments [10].

Interestingly, we also observed a negative correlation between post-load ghrelin levels and height SDS in SGA children. Despite the absence of short stature in this group and the lack of parental height data, this observation is worth noting. This relationship may reflect differences in catch-up growth: children who achieved better linear growth tend to have lower ghrelin concentrations after glucose administration, whereas shorter SGA children may retain higher post-glucose ghrelin as a residual compensatory signal supporting further growth and energy uptake. As a metabolic signal, ghrelin plays an important effect on regulating energy balance during early life growth and development. Ghrelin levels appear to be elevated in SGA infants before catch-up growth but typically decrease once nutritional status and adiposity normalize [5,10]. Recent evidence summarized by Zhang et al. highlights ghrelin as a link between impaired fetal growth, postnatal catch-up growth, and long-term metabolic homeostasis in SGA individuals [10]. Nevertheless, the role of ghrelin in SGA-related metabolic regulation cannot be inferred from circulating concentrations alone. Future studies should examine ghrelin-regulated signaling pathways to better identify SGA individuals at increased metabolic risk and to guide targeted strategies aimed at preventing growth disturbances and insulin-resistance-related disorders later in life [10].

Some limitations of our study should be considered. First, its cross-sectional design precludes conclusions about causality or temporal changes in ghrelin regulation across development. Second, we measured total ghrelin but were unable to assess acylated ghrelin, which is the biologically active fraction; however, total ghrelin directly reflects the levels of unacylated and acylated ghrelin and remains widely used in pediatric studies and correlates with metabolic status. Third, we did not include a control group of children born appropriate to gestational age (AGA), which would have allowed direct comparisons.

Despite these limitations, this study represents the first comprehensive evaluation of ghrelin responses during OGTT in prepubertal SGA children with catch-up growth and provides clinically relevant insights into their metabolic phenotype.

## 4. Materials and Methods

The study protocol was approved by the Bioethics Committee of the Polish Mother’s Memorial Hospital—Research Institute (PMMH-RI), Lodz, Poland (No. 12/2007). All procedures were conducted in accordance with the Declaration of Helsinki and relevant national regulations governing biomedical research involving human participants.

Potential participants were identified using the database of the PMMH-RI in Lodz (which is a 3rd-degree reference center for perinatal care). Parents of children born in five consecutive years with a birth weight below 2500 g and aged 5–9 years at the time of screening were invited to participate. The final study group included children attending PMMH-RI, whose parents provided written informed consent, who met all inclusion criteria and completed an oral glucose tolerance test (OGTT) as part of the study protocol. The inclusion criteria included the following: age, ≥4 and ≤10 years; patient’s height, more than −2.0 SDS with respect to age and sex; born with weight less than −2.0 SDS (SGA). Only full-term children (gestational age ≥ 38 weeks) without severe birth defects or genetic syndromes were included in the study. Therefore, infants born before 38 weeks of gestation and children with birth defects and genetic disorders, as well as those with actual high below −2.0 SDS values (persistent short stature, i.e., without catch-up growth) were excluded from the study.

A detailed physical examination was performed in all participants, and pubertal stage was evaluated according to the Tanner scale. The height of each child was measured using a Harpenden stadiometer and body weight using the scale, followed by the calculation of the BMI. The SDS for height, body weight and BMI was calculated according to the reference values for Polish children [35]. Waist circumference was measured in each child. Obesity was diagnosed when BMI was >+2.0 SDS, and visceral obesity was defined as a waist circumference above the 90th percentile for sex and age [36].Blood pressure (SBP, DBP) was measured in full accordance with current guidelines for arterial pressure assessment in children. All measurements were obtained by an experienced nurse using a standardized protocol. After a 10 min rest in the reclining position, blood pressure was measured with the auscultatory method using the same sphygmomanometer throughout the study. Elevated blood pressure was defined as values exceeding the 90th percentile for sex and height according to Polish pediatric reference charts.

All participants underwent an oral glucose tolerance test (OGTT) following an overnight fast as part of the study protocol to assess physiological metabolic and hormonal responses under standardized conditions. A glucose load of 1.75 g per kilogram of body weight (maximum 75 g) was administered orally. Blood samples were collected at 0, 60, and 120 min for glucose and insulin determination, and at 0 and 120 min for ghrelin measurement. The fasting sample (0 min) was also used for lipid profile assessment. Glucose values ≥ 100 mg/dL (fasting) or ≥140 mg/dL (at 120 min of OGTT) were considered abnormal. Insulin resistance was assessed using the HOMA-IR index: (fasting glucose [mmol/L] × fasting insulin [µIU/mL])/22.5. A HOMA-IR > 2.5 was interpreted as insulin resistance. Additionally, patients were divided into two groups according to HOMA-IR levels, using the 75th percentile of the study population as the cut-off to define lower and higher HOMA-IR.

Total ghrelin was measured using Millipore RIA kit (Linco Research, St. Charles, MO, USA), with the sensitivity range: 100–10,000 pg/mL, the intra-assay CV: 3.3–10.0% and the inter-assay CV: 14.7–17.8%. Plasma glucose was measured by the enzymatic hexokinase method on an automated analyzer. Plasma insulin concentration was measured using the DRG ELISA kit with the sensitivity range: 1.76–100 µIU/mL, the intra-assay CV: 1.8% to 2.6%, and the inter-assay CV: 2.9% to 6.0%.

Statistical analyses were performed using StataNow/BE version 19.5 (StataCorp LLC, College Station, TX, USA). Data were expressed as median (interquartile range, Q1–Q3) and compared using non-parametric tests due to non-normal distribution. Between-group comparisons were assessed with the Mann–Whitney U test for continuous variables and the χ^2^ test for categorical variables. Changes in ghrelin levels during OGTT were evaluated with the Wilcoxon signed-rank test. Correlations were analyzed using Pearson correlation coefficients. Multivariable linear regression was performed to identify independent predictors of fasting ghrelin, 120 min ghrelin, and ghrelin suppression. A *p*-value < 0.05 was considered statistically significant.

## 5. Conclusions

Ghrelin concentrations in SGA children decreased significantly during OGTT, with obese participants showing lower fasting and post-load levels as well as blunted suppression. Both fasting and post-load ghrelin levels, as well as ghrelin suppression, were associated with insulin-related parameters and indices of adiposity. Notably, as shown in multivariable analyses adjusted for age, sex, and BMI SDS, ghrelin measures remained independently associated with insulin-related parameters. Together, these findings suggest that disturbances in ghrelin-related signaling may be involved in the metabolic alterations observed in SGA children.

## Figures and Tables

**Table 1:** Characteristics of the study group (SGA) divided by obesity status.

Variable	Non-Obese (*n* = 59)	Obese (*n* = 39)	*p* Value
Age (years)	6.18 (5.28–7.47)	7.85 (7.23–9.04)	**<0.001**
Sex (N girls/boys)	24/35	16/23	**0.97**
Birth weight (g)	2300 (2120–2400)	2250 (2150–2300)	0.5
Gestational age (weeks)	38 (38–39)	38 (38–39)	0.20
Birth weight SDS	−2.4 (−2.63–−2.21)	−2.4 (−2.69–−2.16)	0.78
Height (cm)	118 (112–127)	132.5 (123.6–140.5)	**<0.001**
Height SDS	0.00 (−0.42–0.60)	0.65 (0.11–1.31)	**<0.001**
BMI (kg/m^2^)	17.29 (16.09–18.94)	23.41 (21.12–26.30)	**<0.001**
BMI SDS	1.00 (0.40–1.61)	3.10 (2.21–4.08)	**<0.001**
Waist circumference (cm)	54 (50.5–56.0)	65 (58.5–69.5)	**<0.001**

Values are expressed as median (Q1–Q3), *p* < 0.05 is bolded.

**Table 2:** Glucose, insulin, HOMA-IR and ghrelin concentrations at baseline (0′) and after 60′ and 120 min during OGTT in SGA children according to obesity status.

Parameter	Non-Obese (*n* = 59)	Obese (*n* = 39)	*p* Value
Glucose 0′ (mg/dL)	82 (75–88)	83 (80–86)	0.5
Glucose 60′ (mg/dL)	106 (91–126)	107 (87–132)	0.95
Glucose 120′ (mg/dL)	106 (98–113)	102 (91–115)	0.41
Insulin 0′ (µIU/mL)	2.22 (1.50–4.39)	4.56 (2.28–7.56)	**<0.001**
Insulin 60′ (µIU/mL)	12.5 (8.41–19.4)	24.6 (11.5–35.7)	**0.004**
Insulin 120′ (µIU/mL)	12.0 (8.94–18.3)	25.2 (16.5–38.1)	**<0.001**
HOMA-IR	0.46 (0.30–0.97)	0.90 (0.47–1.55)	**<0.001**
Fasting ghrelin 0′ (pg/mL)	2181.7 (1537.6–3648.2)	1388.5 (1153.1–2190.9)	**<0.001**
Ghrelin 120′ (pg/mL)	1303.2 (1046.4–1907.6)	957.2 (781.5–1402.1)	**0.005**
Δ Ghrelin 0–120′ (pg/mL)	688.5 (370.3–1597.1)	422.4 (146.5–789.0)	**0.003**

Values are expressed as median (Q1–Q3), *p* < 0.05 is bolded.

## Data Availability

The data presented in this study are available on request from the corresponding author.

## Abbreviations

The following abbreviations are used in this manuscript:

AGA	Appropriate for gestational age
BMI	Body mass index
BP	Blood pressure
DBP	Diastolic blood pressure
GH	Growth hormone
GI	Glycemic index
GHSR	Growth hormone secretagogue receptor
GOAT	Ghrelin O-acyl transferase
HDL	High-density lipoprotein
HOMA-IR	Homeostatic model assessment of insulin resistance
IGF-I	Insulin-like growth factor I
LDL	Low-density lipoprotein
OGTT	Oral glucose tolerance test
SBP	Systolic blood pressure
SDS	Standard deviation score
SGA	Small for gestational age
